# Editorial: Cell network in antitumor immunity of pediatric and adult solid tumors

**DOI:** 10.3389/fimmu.2023.1246695

**Published:** 2023-07-06

**Authors:** Silvia Pesce, Ombretta Melaiu

**Affiliations:** ^1^Department of Experimental Medicine and Centre of Excellence for Biomedical Research, University of Genoa, Genoa, Italy; ^2^Department of Clinical Sciences and Translational Medicine, University of Rome Tor Vergata, Rome, Italy

**Keywords:** tumor microenvironment (TME), tumor-infiltrating immune cells, biomarkes, tumor immunity, immunotherapy

The tumor microenvironment (TME) is a highly structured ecosystem composed of cancer cells and a variety of non-cancer cells embedded in an altered and vascularized extracellular matrix. A rich diversity of immune cells, cancer-associated fibroblasts (CAFs), and endothelial cells, previously considered only bystanders in tumorigenesis, are now recognized as key players in neoplasms and thus represent attractive targets for prognostic and therapeutic purposes (1). Tumor progression, for example, is associated with a decrease in cytotoxic T and NK cells, an increase in exhausted CD8^+^ T cells (2), immunosuppressive CD4^+^ FOXP3^+^ Tregs (3), and regulatory B cells (4). In contrast, dendritic cells (DCs) show defective maturation and function (5). Along with immune populations, CAFs are a dominant component of many cancer types. The activation of CAFs in the TME can be the result of several mechanisms, including exposure to inflammatory mediators, changes in extracellular matrix (ECM) composition and stiffness, and altered metabolites (6). In this regard, the crucial role of deregulated metabolic demands in generating a TME supportive of neoplastic progression is becoming increasingly clear (7). Importantly, all these aspects have implications for the efficacy of immunotherapy (as well as chemotherapy and radiotherapy), and a major effort is underway to identify combinatorial therapeutic strategies that take advantage of inhibitors and/or modulators of the various TME components.

This Research Topic was devised to update our current knowledge on the complex interconnectedness of the TME and its influence on disease progression and response to therapy. We have collected a series of articles that provide us with in-depth evaluations of the role of different types of immune and stromal cells in the control of solid tumors, novel immunotherapeutic strategies, and multi-omics approaches that offer further insights into this field. In brief, this Research Topic includes seven original research papers, three case reports, one perspective and two reviews of the current literature.

In recent years, a growing number of studies have investigated the key characteristics of NK and T cells in different disease settings. In this context, the work of Caforio et al. has identified Che1 as a key protein able to promote the viability of tumor cells, but also the expression of the Nectin-1 ligand, resulting in an impaired killing activity of NK cells. These results suggest how the identification of targets with a dual function, i.e., cancer promoter, and modulator of the immune response, could lead to much more potent therapeutic strategies for eradicating a malignancy. In line with this, Bergantini et al. better explored the pathogenesis of sarcoidosis by analyzing the frequency and phenotype of NK and T cells in two different districts: bronchoalveolar lavage (BAL) and peripheral blood (PB). The authors showed that compared to PB, BALs were mainly infiltrated by a subset of CD56brightCD16neg NK cells and of memory effector T cells. In addition, the more mature BAL-NK cell subset (CD56dim/negCD16^+^) expressed higher levels of PD1 and activation markers, such as NKp44, CD69 and CD25.

The partially unsuccessful use of immune checkpoint inhibitors (ICIs) in patients with poorly immunogenic neoplasms and highly immunosuppressive TME (8) has led to a growing interest in better characterizing the role of DCs, heterogeneous population playing a central role in the activation and regulation of all immune responses. A detailed evaluation of glioma infiltrating DC subpopulations and their activating/tolerogenic profile was performed by Carenza et al. Their results showed a significant reduction of circulating DCs and a concomitant intratumoral recruitment of all DC subpopulations, which were however functionally impaired. Their drastic functional impairment was even more evident in glioma patients undergoing perioperative steroid treatment, usually administered to control peritumoral edema. This suggests the use of alternative therapeutic strategies to control this symptom.

It is well known that also the intratumoral spatial organization of immune cells and their crosstalk with other cellular components play a crucial role in determining prognosis and response to immunotherapy in cancer patients (9). Timperi et al. reviewed the suppressive crosstalk between newly identified macrophages and CAF subpopulations in a variety of solid tumors and proposed targets that could be used as potential novel therapeutic approaches. Concurrently, the importance of tertiary lymphoid structures (TLS) is another area of great interest (10). Two articles in our Research Topic addressed their role in breast and lung cancers, respectively. A first remarkable observation concerns the differential impact of TLSs maturation status on tumour progression. Indeed, a high number of mature TLSs, as shown by Wang et al., is associated with a better prognosis of breast cancer patients, suggesting that TLSs are privileged sites for local lymphocyte differentiation and antigen presentation. In contrast, Zhao et al. associated the abundance of immature TLS with lack of response to immunotherapy in a lung adenocarcinoma (LUAD) patient, characterized by high FOXP3^+^ regulatory T cells and increasing levels of the circulating checkpoint proteins BTLA, TIM-3, LAG-3, PD-1, PD-L1, and CTLA4. Consistent with previous findings, Cai et al., in evaluating the efficacy of neoadjuvant chemo-immunotherapy compared with chemotherapy alone, showed that only patients with increased TLS and concomitant infiltration of B and T cells were able to undergo major pathologic response (MPR) when treated with chemotherapy alone. In the remaining cases, the addition of ICIs to chemotherapy was associated with a significantly higher rate of MPR together with a major abundance of CD8^+^ T cells in the tumor stroma and M1 macrophage density in the tumor center. Interestingly, the importance of adding ICIs has been demonstrated not only in the neoadjuvant setting, but also after multiple lines of adjuvant treatment, as reported by Zhang et al., in a patient with small cell lung cancer.

In cancer immunotherapy, in addition to ICIs designed to augment natural immune responses, other types of neoplasms are being treated with chimeric antigen receptors (CARs), designed to induce new immune responses directed against tumor-expressed targets (11). For CAR T cells to be effective, bridging therapy is often required (12). Saldi et al. demonstrated that an extended radiotherapy approach is an excellent strategy to enhance the effect of CD19-directed CAR T-cell therapy, leading to a complete remission of the disease in a patient with relapsed/refractory diffuse large B-cell lymphoma. However, since the use of CAR T cells can lead to graft-versus-host disease (GvHD) and cytokine release syndrome, there is increasing interest in the engineering of NK cells, which have a higher safety profile. To date, NK cells have been engineered against various CARs or the chimeric NKG2D receptor and have shown promising results in preclinical and clinical models. In addition to NKG2D, other activating receptors may also yield encouraging responses. For example, Cifaldi et al. proposed the use of the never-before-explored DNAM-1 chimeric receptor engineered-NK cells. The authors provide a rationale predicting that this therapeutic tool has several strengths to consider: first and foremost, the fact that, unlike other constructs, NK cells engineered for DNAM-1 are able to specifically target tumor cells that express high levels of PVR and Nectin-2, while tolerating normal cells that usually express low levels of these ligands.

These latest studies highlight another important need: quickly identifying patients who may respond to one treatment over another. Many factors influence for example the effectiveness of immunotherapy, and few biomarkers have been developed so far to assess its benefit accurately (11). In this context, Huang et al. applied integrated analysis to develop a four genes-prognostic signature, called LATPS, for LUAD patients. The LATPS-low subgroup had better survival, and a greater chance of benefiting from immunotherapy, thus representing a promising prognostic tool with clinical utility. Similarly, by studying the role of lactate in LUAD TME, Shang et al. established a gene signature called “LaSig” that can predict survival and response to immunotherapy as well as to cisplatin, erlotinib, gemcitabine and vinblastine in these patients. Using single cell RNAseq data, Xie et al. showed that immune, stromal, and tumor cells of colorectal cancer patients share similar lipid metabolism during their terminal differentiation, that confers an immunosuppressive microenvironment. In addition, through the integration of scRNA-seq and mass-RNA-seq data, they built an immune and clinical risk model with high prognostic power. Finally, Rozenberg et al. reviewed the pathological mechanisms directly involved in the formation and pathogenesis of circulating heterotypic tumor cells (CTCs) emerging as prognostic and therapeutic markers in metastatic malignancies.

In summary, the papers included in this Research Topic represent the latest advances in the field of immuno-oncology. Based on these studies, we can believe and trust that in-depth exploration of the TME promises to advance tumor treatment research in the next decade.

## Author contributions

OM and SP: drafted the article, provided critical inputs, and corrected the manuscript. All authors contributed to the article and approved the submitted version.
